# LncRNA *AERRIE* Is Required for Sulfatase 1 Expression, but Not for Endothelial-to-Mesenchymal Transition

**DOI:** 10.3390/ijms22158088

**Published:** 2021-07-28

**Authors:** Tan Phát Pham, Anke S. van Bergen, Veerle Kremer, Simone F. Glaser, Stefanie Dimmeler, Reinier A. Boon

**Affiliations:** 1 Department of Physiology, Amsterdam UMC, Vrije Universiteit Amsterdam, 1081 HV Amsterdam, The Netherlands; t.pham1@amsterdamumc.nl (T.P.P.); a.vanbergen@amsterdamumc.nl (A.S.v.B.); v.kremer@amsterdamumc.nl (V.K.); 2 Institute of Cardiovascular Regeneration, Goethe University, 60590 Frankfurt am Main, Germany; glaser@med.uni-frankfurt.de (S.F.G.); dimmeler@em.uni-frankfurt.de (S.D.); 3 German Center for Cardiovascular Research DZHK, Partner Site Frankfurt Rhine-Main, 60590 Frankfurt am Main, Germany

**Keywords:** Non-coding RNA, EndMT, endothelial cells

## Abstract

Endothelial cells can acquire a mesenchymal phenotype through a process called Endothelial-to-Mesenchymal transition (EndMT). This event is found in embryonic development, but also in pathological conditions. Blood vessels lose their ability to maintain vascular homeostasis and ultimately develop atherosclerosis, pulmonary hypertension, or fibrosis. An increase in inflammatory signals causes an upregulation of EndMT transcription factors, mesenchymal markers, and a decrease in endothelial markers. In our study, we show that the induction of EndMT results in an increase in long non-coding RNA *AERRIE* expression. JMJD2B, a known EndMT regulator, induces *AERRIE* and subsequently *SULF1*. Silencing of *AERRIE* shows a partial regulation of *SULF1* but showed no effect on the endothelial and mesenchymal markers. Additionally, the overexpression of *AERRIE* results in no significant changes in EndMT markers, suggesting that *AERRIE* is marginally regulating mesenchymal markers and transcription factors. This study identifies *AERRIE* as a novel factor in EndMT, but its mechanism of action still needs to be elucidated.

## 1. Introduction

Endothelial-to-mesenchymal transition (EndMT) is a specific cellular process in which endothelial cells change their phenotype towards mesenchymal cells. It is a dynamic development in which endothelial cells transform into a more migratory and invasive state that results in increased motility and loss of barrier function and cell junctions [1]. EndMT is essential during the embryonic developmental stage and was first described in the development of the heart valves and pulmonary artery [2]. Recently, partial EndMT has been described as a process involved in the onset of angiogenesis, vessel, and heart development [3]. Failure of EndMT results in failure of valve formation, whereas induction of EndMT results in thicker pathological valve formation [4,5]. Interestingly, EndMT is described as being involved not only in embryonic development, but also in pathological processes as well. EndMT has been reported to contribute to many cardiovascular diseases (CVDs) related to aging, such as pulmonary arterial hypertension, atherosclerosis, inflammation, and fibrosis, due to structural and functional changes in endothelial cells [6,7,8,9]. Since the molecular defects in pathological EndMT are poorly understood, understanding the mechanism of EndMT may reveal novel opportunities for CVD intervention.

EndMT is induced by various growth factors and cytokines. Transforming growth factor-β (TGF-β) and interleukin-1β (IL-1β), for example, are known stimulators of EndMT, while the TGF-β superfamily is the major regulator [10,11,12,13,14,15]. This superfamily is comprised of all the TGF-β isoforms, bone morphogenetic proteins (BMPs), activins, Smad proteins, and more [16]. These modulators regulate the expression of many transcription factors, such as SNAIL, Slug, ZEB1, and ZEB2 [17,18,19]. Subsequently, these transcription factors increase mesenchymal markers such as α-smooth muscle actin (α-SMA), smooth muscle 22 (SM22), and calponin (CNN1), and decrease endothelial markers such as vascular endothelial cadherin (VE-cadherin), and platelet endothelial cell adhesion molecule-1 (PECAM-1). Furthermore, long non-coding RNAs (lncRNAs) are known to be involved in EndMT, and elucidating their role will improve our understanding of CVDs [20].

LncRNAs are transcripts that do not code for a protein and are longer than 200 nucleotides. More than 300,000 lncRNAs have been identified in humans and animals, but only a few of them are characterized in detail. Depending on their location in the cell, several functions have been identified. In the nucleus, lncRNAs regulate transcription by binding to transcription factors and chromatin modifiers [21,22]. In the cytosol, they are associated to mRNAs or miRNAs to modulate their stability or influence the transcriptome, respectively [23,24,25]. Many lncRNAs have been identified as being involved in CVDs; however, their function and mechanism are still not fully understood [26]. For instance, the lncRNA H19 has been associated with myocardial infarction, cardiac hypertrophy, and aging via inhibition of STAT3 signalling [27,28,29]. Moreover, The LncRNA *MEG3* is described as promoting myocardial infarction and fibrosis by regulating *TLR4* [30,31,32,33]. Furthermore, the lncRNA *Mhrt* is downregulated in pressure-overloaded hearts and is able to prevent cardiomyopathy progression [34]. Finally, lncRNA *Chrf* is upregulated in cardiac hypertrophy by targeting miR-489 and *Myd88* in cardiac hypertrophy [35].

The lncRNA *AERRIE* (*LINC01013*) is expressed in endothelial cells and is shown to be regulated by aging [36]. *AERRIE* is associated with the RNA-binding protein YBX1, and both act together as important factors in DNA damage signaling and repair [36]. In hepatocellular carcinoma stem cells, *AERRIE* regulates spheroid and colony formation, proliferation, and stemness markers, while in stem cells from apical papilla, DLX5 and HOXC8 enhance chondrogenic differentiation via *AERRIE* [37,38]. Interestingly, in an older study by Chung et al. *AERRIE* was found to regulate the invasiveness of human anaplastic large-cell lymphomas via epithelial-to-mesenchymal transition (EMT), an analog of EndMT [39].

In our study, the lncRNA *AERRIE* is expressed in endothelial cells, and we identified that *AERRIE* is upregulated by EndMT. We show that the knockdown of *AERRIE* is not sufficient to reverse EndMT. Moreover, the overexpression of *AERRIE* does not induce EndMT. Interestingly, JMJD2B, a known regulator of EndMT [40], regulates *AERRIE* and subsequently regulates *SULF1*. However, *AERRIE* is only partially required for *SULF1* expression.

## 2. Results

### 2.1. Endothelial Cells Stimulated with TGF-β2 and IL1β Undergo EndMT

**Figure 1:**
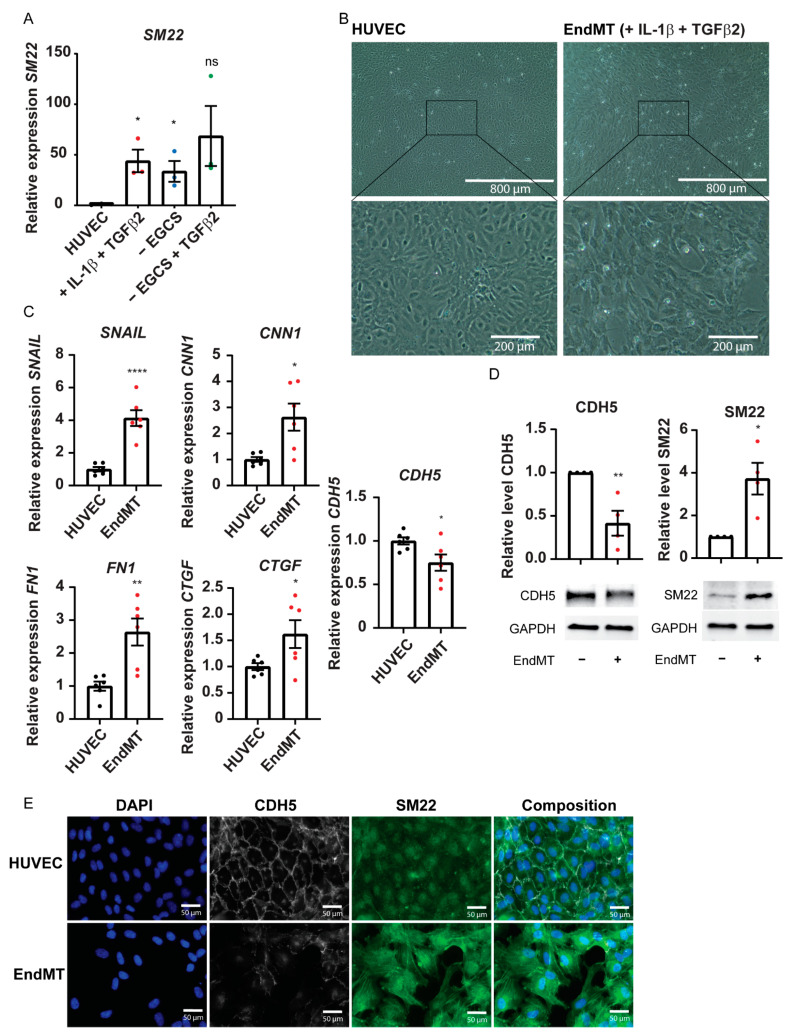
Successful Endothelial-to-Mesenchymal Transition (EndMT) of HUVECs by IL-1β and TGF-β2. (**A**–**E**) HUVECs were stimulated with IL-1β and TGF-β2 for 72 h to induce EndMT. EndMT condition is defined as the HUVECs treated with IL-1β and TGF-β2. (**A**) Expression levels of SM22 were measured by real-time quantitative PCR (RT-qPCR). Expression values are relative to the basal HUVEC condition and normalized to RPLP0 mRNA (*n* = 3). (**B**) Brightfield images from human umbilical vein endothelial cells (HUVECs) and endothelial-to-mesenchymal transition (EndMT) cells. HUVECs were exposed to IL-1β and TGF-β2 for 72 h to induce EndMT. (**C**) Expression levels of EndMT markers (SM22, SNAIL, CNN1, FN1, and CTGF) were measured by real-time quantitative PCR (RT-qPCR). Expression values are relative to the HUVEC condition and normalized to RPLP0 mRNA (*n* = 6). (**D**) VE-Cadherin (Endothelial marker) and SM22 (Mesenchymal marker) protein levels were analyzed by Western blotting (*n* = 3). GAPDH protein levels were used as a loading control. (**E**) Cell morphology and monolayer structure were analyzed by immunofluorescence. Nuclei were visualized with DAPI on the 405 nm channel. Endothelial cell junctions were visualized with VE-Cadherin staining on the 555 nm channel. Mesenchymal marker SM22 was visualized on the 488 nm channel. * *p* < 0.05; ** *p* < 0.01; **** *p* < 0.0001; ns, not statistically significant.

LncRNA *AERRIE* is previously described as being regulated by aging, and is specifically involved in DNA repair in endothelial cells [36]. *AERRIE* is regulated by many of the hallmarks of aging, such as shear stress and EndMT [36]. Therefore, we wanted to assess whether *AERRIE* contributes to EndMT. First, we evaluated which stimulant would efficiently induce EndMT in HUVECs. For this purpose, we stimulated human umbilical vein endothelial cells (HUVECs) with three different conditions: TGF-β2 and IL1β, removing Endothelial Cell Growth Supplement (ECGS), and removing ECGS while stimulating with TGF-β2 for 72 h (Figure 1A). The removal of ECGS or the treatment with IL-1β and TGF-β2 showed the most stable increase in *SM22* expression. Therefore, subsequent experiments were performed with stimulation with IL-1β and TGF-β2, as an EndMT condition. The cultured HUVECs underwent morphological changes with mesenchymal features (Figure 1B). The endothelial cells lost their cobblestone-like structure, and formed a spindle-shaped elongated structure together with gap formation between the cells. At the transcriptional level, we observed a significant increase in mesenchymal marker expressions (Figure 1C). RNA expression of mesenchymal markers *CNN1*, *FN1, CTGF,* and *SNAIL* were upregulated. We also observed on Western blot that the protein level of the endothelial marker VE-Cadherin was decreased while the SM22 protein level was increased, showing that the HUVECs lost their endothelial properties and gained mesenchymal features (Figure 1D). To confirm these findings, we confirmed the mesenchymal transition with immunofluorescence microscopy. HUVECs showed proper VE-Cadherin junction staining and a low SM22 signal, as well as a cobble-like structure before stimulation with TGF-β2 and IL1β. After stimulation, a reduction in VE-Cadherin, induction of SM22, and loss of the cobble-like structure was observed (Figure 1E). Altogether, we successfully induced EndMT in HUVECs using TGF-β2 and IL1β stimulation. We observed that the endothelial monolayer is disturbed by the loss of the endothelial specific cobble-like structure, loss of cell junctions, and increase in spindle-like structures. In addition, the number of cells decreased, probably due to a loss of adhesion caused by EndMT.

### 2.2. Inhibition of lncRNA AERRIE Does Not Regulate EndMT Markers

**Figure 2:**
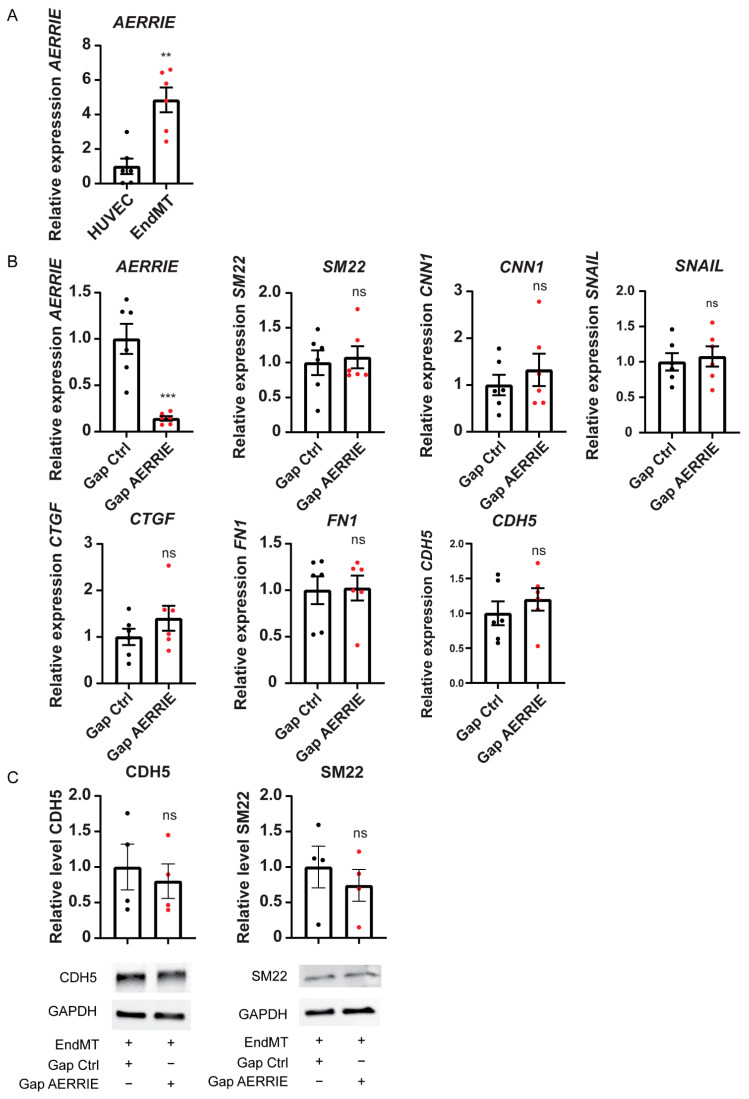
*AERRIE* is upregulated by EndMT but has no direct influence on EndMT induction. (**A**) Expression level of *AERRIE* was measured by real-time quantitative PCR (RT-qPCR). Expression values were relative to the unstimulated HUVEC condition and normalized to *RPLP0* mRNA (*n* = 6). (**B**,**C**) EndMT HUVECs were treated with gapmeR (gap) targeting *AERRIE* or a respective control. (**B**) Expression level of *AERRIE*, EndMT markers (*SM22*, *CNN1*, *SNAIL*, *CTGF*, *FN1*), and endothelial marker VE-Cadherin were measured by real-time quantitative PCR (RT-qPCR). Expression values are normalized to *RPLP0* mRNA (*n* = 6). (**C**) VE-Cadherin (Endothelial marker) and SM22 (Mesenchymal marker) protein levels were analyzed by Western blotting (*n* = 4). GAPDH protein levels were used as a loading control. ** *p* < 0.01; *** *p* < 0.001; ns, not statistically significant.

HUVECs undergoing EndMT by stimulation with TGF-β2 and IL1β displayed increased expression of *AERRIE,* as shown in our previous study (Figure 2A) [36]. To assess whether EndMT is regulated by *AERRIE* we silenced *AERRIE* with LNA-GapmeRs. LNA-GapmeRs are DNA oligos that specifically bind to their target RNAs, which results in degradation by RNAse H in the nucleus [41]. In this study, the use of LNA-GapmeRs showed a successful downregulation of *AERRIE* (Figure 2B). However, the inhibition of *AERRIE* did not result in a change in RNA expression of the EndMT markers *SM22*, *SNAIL*, *CTGF*, *CNN1,* and *FN1* (Figure 2B). Moreover, protein levels of SM22 and VE-Cadherin did not change upon knockdown of *AERRIE* (Figure 2C), suggesting that *AERRIE* does not regulate EndMT markers.

### 2.3. AERRIE Does Not Regulate Barrier Function in HUVECs Undergoing EndMT

**Figure 3:**
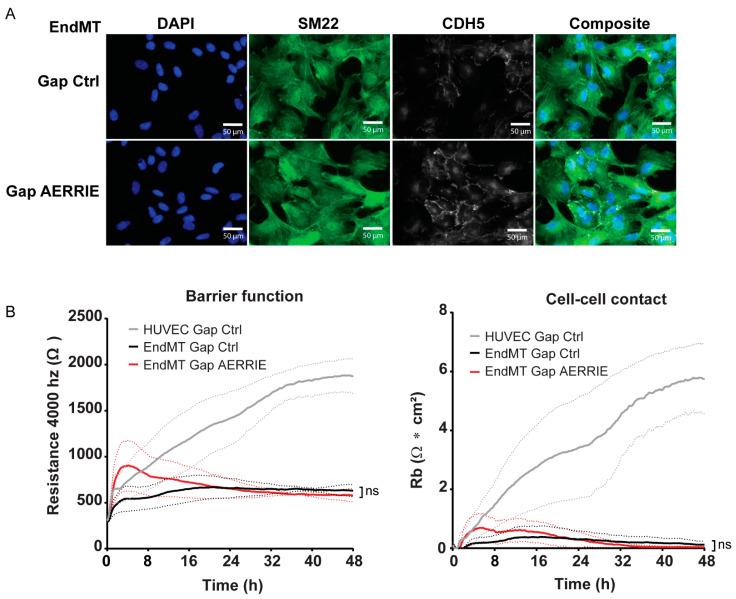
Knockdown of *AERRIE* does not change morphology and barrier function of EndMT-stimulated HUVECs. (**A**,**B**) EndMT HUVECs were treated with gapmeR (gap) targeting *AERRIE* or a respective control. (**A**) Cell morphology and monolayer structure were analyzed by immunofluorescence. Nuclei were visualized with DAPI on the 405 nm channel. Endothelial cell junctions were visualized with VE-Cadherin staining on the 555 nm channel. Mesenchymal marker SM22 was visualized on the 488 nm channel. (**B**) Electric cell-substrate impedance sensing (ECIS) was performed to measure the barrier function of the endothelial and mesenchymal monolayer. The resistance of the monolayer was determined after 48 h (*n* = 3). Gap Ctrl HUVECs were used as control.

As previously shown, HUVECs undergoing EndMT contain higher SM22 levels compared to unstimulated cells (Figure 1B,C). To assess whether SM22 or VE-Cadherin localization is regulated by *AERRIE*, we performed an immunostaining of EndMT cells (Figure 3A). Upon the knockdown of *AERRIE,* no morphological changes were observed in comparison to the transfected GapmeR control (Gap Ctrl). The spindle-like morphology was unaltered or the cell–cell junctions were equally disturbed in both conditions, suggesting a loss in barrier function. Similarly, the signal intensity of SM22 or VE-Cadherin did not change in comparison to the control, confirming that the protein levels were not significantly altered. To determine the stability of the barrier, we measured the resistance of the endothelial monolayer by Electric Cell-substrate Impedance Sensing (ECIS). In these experiments, ECIS was performed with Gapmer Ctrl-treated HUVECs as a control, and EndMT-treated HUVECs with and without *AERRIE* (Figure 3B). HUVECs undergoing EndMT lost resistance on ECIS, from 1800 Ohm to 500 Ohm, after 48 h of measurement. The knockdown of *AERRIE* did not change the amount of resistance in ECIS compared to the Gap Ctrl. In addition, the cell–cell contacts showed a similar pattern where HUVECs maintained a high cell–cell contact, whereas the EndMT control and *AERRIE* knockdown condition were impaired. Our previous work showed that the knockdown of *AERRIE* in a non-EndMT condition altered the resistance in ECIS [36]. Altogether, the data confirm that *AERRIE* is not required for EndMT.

### 2.4. Overexpression of AERRIE Does Not Affect EndMT Marker Levels or Barrier Function

**Figure 4:**
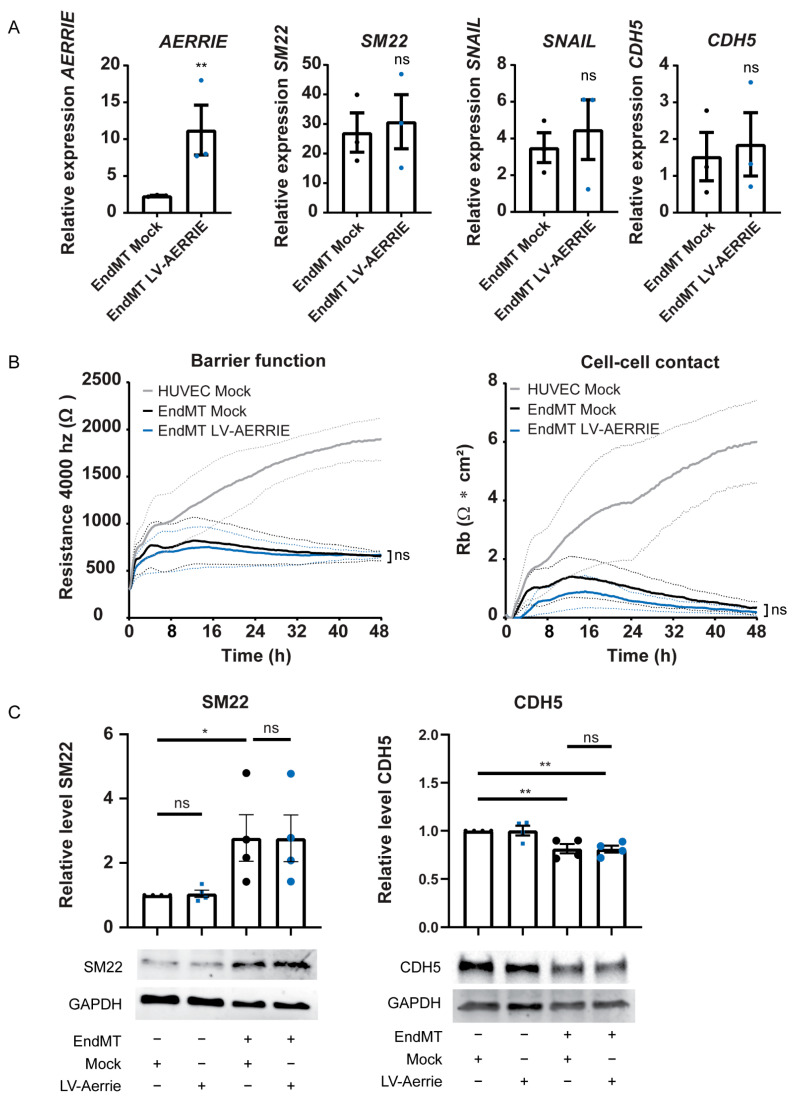
Lentiviral overexpression of *AERRIE* does not change barrier function, mesenchymal marker RNA expression, or mesenchymal and endothelial protein levels. (**A**–**C**) EndMT induced HUVECs were treated with lentivirus (LV) for *AERRIE* overexpression or a respective control. (**A**) Expression level of *AERRIE*, *SM22*, and *SNAIL* were measured by real-time quantitative PCR (RT-qPCR). Expression values were relative to the mock control condition and normalized to *RPLP0* mRNA (*n* = 3). (**B**) Electric cell-substrate impedance sensing (ECIS) was performed to measure the barrier function of the endothelial and mesenchymal monolayer. The resistance of the monolayer was determined after 48 h (*n* = 3). HUVECs with a mock control were used as control to EndMT HUVECs. (**C**) VE-Cadherin (Endothelial marker) and SM22 (Mesenchymal marker) protein levels were analyzed by Western blotting (*n* = 3). GAPDH protein levels were used as a loading control. * *p* < 0.05; ** *p* < 0.01; ns, not statistically significant.

To examine whether EndMT markers are inhibited by *AERRIE*, we overexpressed *AERRIE* via lentiviral transduction. The overexpression of *AERRIE* was successfully performed with a 10-fold increase in *AERRIE* expression in HUVECs undergoing EndMT (Figure 4A). However, the treatment did not alter the expression levels of the EndMT markers *SM22*, *SNAIL*, and VE-Cadherin. Furthermore, the effect of *AERRIE* overexpression was determined by ECIS, but no effect on the resistance of the cell layer was observed (Figure 4B). No differences were observed in barrier function resistance, cell–cell contact, or cell–matrix interaction compared to the Mock control. A possible explanation for this finding is that the resistance of the cell layer could not be reduced further than the minimum detection of the instrument, resulting in no further decrease in the observed resistance. Similarly, we did not observe protein level changes in SM22 and VE-Cadherin upon overexpression of *AERRIE* (Figure 4C). Altogether, the data show that a reduction in or induction of *AERRIE* does not influence EndMT, suggesting that the contribution of *AERRIE* to EndMT is marginal.

### 2.5. AERRIE Is Regulated by the EndMT Regulator JMJD2B and Is Required for SULF1 Expression

**Figure 5:**
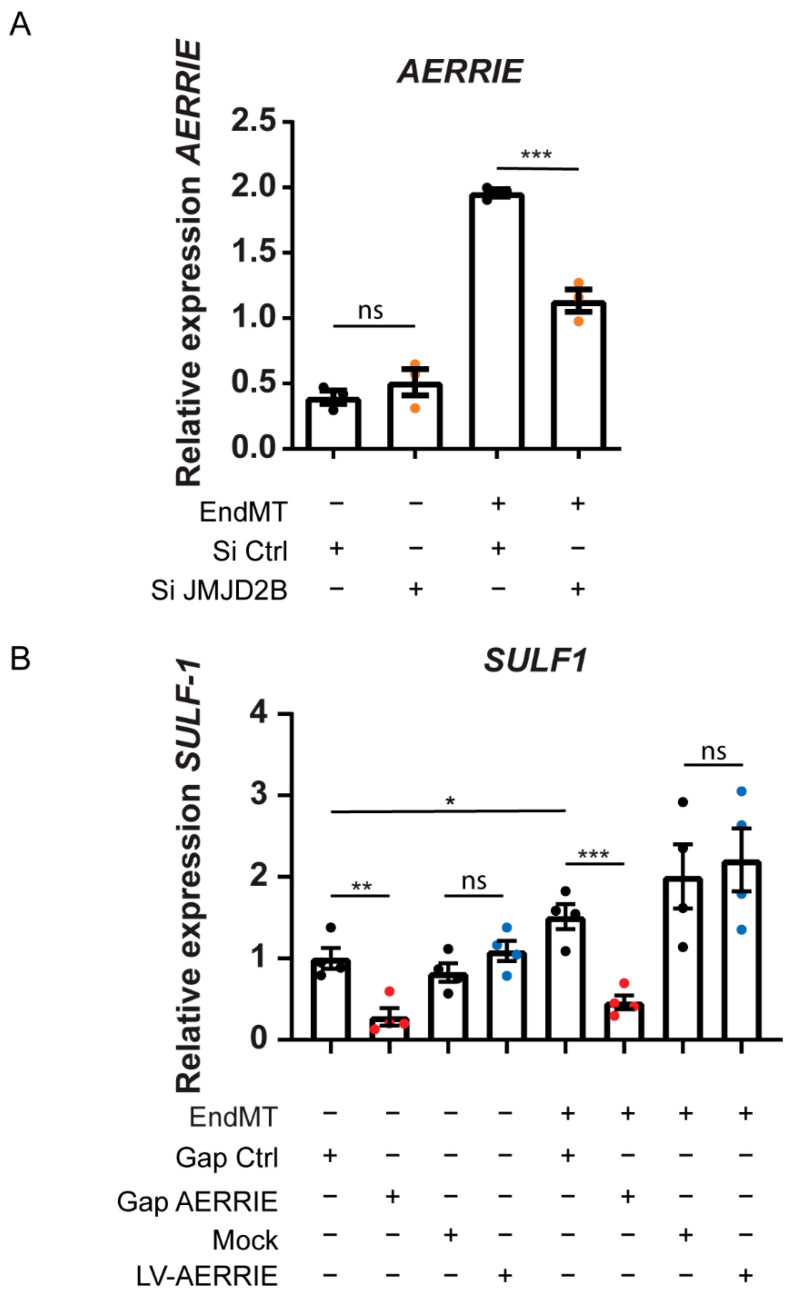
*AERRIE* is regulated by *JMJD2B* in EndMT and subsequently regulates *SULF1*. (**A**,**B**) HUVECs were stimulated with IL-1β and TGF-β2 for 72 h to induce EndMT. Cells were also treated with gapmer (gap) targeting *AERRIE*, siRNA (si) targeting *JMJD2B*, lentivirus (LV) for *AERRIE* overexpression or a respective control. (**A**) Expression level of *AERRIE* was measured by real-time quantitative PCR (RT-qPCR). Expression values were normalized to *RPLP0* mRNA (*n* = 3). (**B**) Expression level of *SULF1* was measured by real-time quantitative PCR (RT-qPCR). Expression values were relative to HUVEC gap control and normalized to *RPLP0* mRNA (*n* = 4). * *p* < 0.05; ** *p* < 0.01; *** *p* < 0.001; ns, not statistically significant.

**Figure 6:**
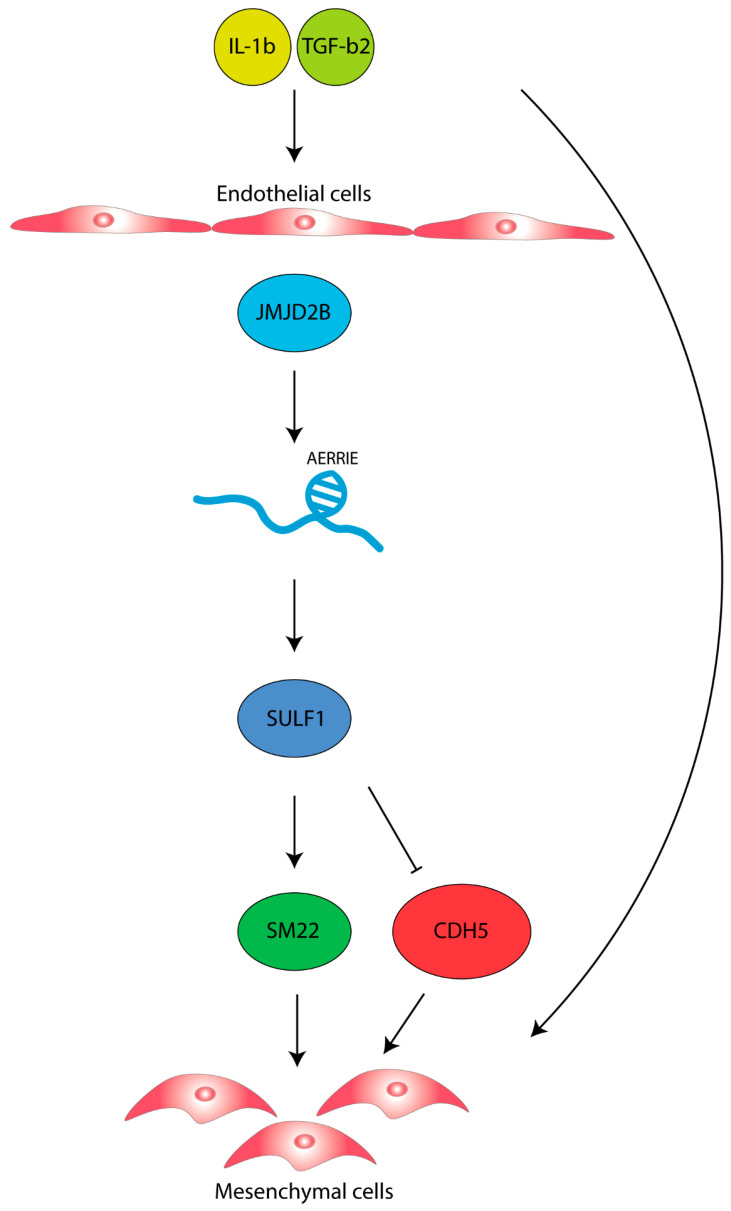
Schematic overview of the function of lncRNA *AERRIE*, JMJD2B and SULF1 in EndMT. *AERRIE* is regulated by IL-1β and TGF-β2 stimulation and by the EndMT inducer JMJD2B resulting in endothelial cells transitioning to mesenchymal cells.

To understand how *AERRIE* is regulated by EndMT, we examined a known EndMT regulator called JMJD2B [40]. JMJD2B is a histone demethylase of the repressive histone mark H3K9me3 and coordinates the methylation of the active histone mark H3K4me3 [42,43]. In endothelial cells, JMJD2B controls *TGF-β2* expression and subsequently alters endothelial morphology and barrier function [40]. To assess whether JMJD2B regulates *AERRIE,* we silenced *JMJD2B* with siRNA. Silencing *JMJD2B* did not result in a change in the expression levels of *AERRIE* compared to the siRNA control in HUVECs (Figure 5A). Interestingly, when *JMJD2B* was silenced in EndMT-stimulated HUVECs, *AERRIE* was significantly downregulated, suggesting that *AERRIE* is regulated by JMJD2B in EndMT. Furthermore, JMJD2B is described as being a transcriptional regulator for *SULF1* [33]. SULF1 is a heperan sulfate endosulfatase that removes sulfate groups from proteoglycans [44]. Modification of the proteoglycans is important for protein interaction and cytokine signaling [45,46]. SULF1 has been shown to regulate TGF-β, FGF, and VEGF signaling [40,47,48]. For this reason, we assessed whether *AERRIE* wass required for *SULF1* expression. Interestingly, *SULF1* was repressed upon silencing *AERRIE* (Figure 5B), under both basal and EndMT conditions. Overexpression of *AERRIE* did not affect *SULF1* expression, suggesting that endogenous *AERRIE* levels are not the rate-limiting factor determining *SULF1* expression. In summary, we observed that the stimulation of HUVECs with IL1β and TGF-β2 induces EndMT and *AERRIE*. However, *AERRIE* does not have a direct effect on EndMT. In parallel, *SULF1* expression requires *AERRIE*, which suggests that *AERRIE* may have an indirect role in EndMT (Figure 6).

## 3. Discussion

CVD is common in the older population and we observed in our previous study that *AERRIE* is regulated by aging. Here, we investigated the role of *AERRIE* in EndMT, which is one of the hallmarks of aging [49]. We demonstrated that lncRNA *AERRIE* is regulated by IL-1β- and TGF-β2-induced EndMT. Loss or overexpression of *AERRIE* did not alter the EndMT phenotype. Altering the expression of *AERRIE* resulted in no changes in the expression of mesenchymal or endothelial markers, and no morphological or functional changes. Barrier function, cell–cell contact, and cell–matrix interaction resistances maintained equal levels, and SM22 and VE-Cadherin protein localization did not differ. Interestingly, JMJD2B, a known EndMT-regulator, has been shown to regulate *SULF1* [40]. In this study, we showed that *JMJD2B* regulates *AERRIE,* and the downregulation of *AERRIE* regulates *SULF1* expression.

Endothelial cells can be triggered to undergo EndMT under certain pathological conditions. It has been demonstrated, via genetic labeling of ECs and disease animal models, that EndMT is involved in wound healing, atherosclerosis, and pulmonary arterial hypertension [6,7,50]. Several studies have shown that blocking EndMT can prevent disease progression. For instance, inhibitors of the EndMT signaling pathways, such as ALK2 kinase inhibitors, have been designed to halt EndMT. This has shown promising results in the prevention of pathological progression [51,52]. Furthermore, previous studies have shown that EndMT can be blocked by certain lncRNAs. lncRNA *MALAT1* for instance, reduces EndMT progression via miR-145 regulation [20]. Moreover, lncRNA *GATA6-AS* can regulate EndMT by interacting with the epigenetic regulator LOXL2. This interaction results in the regulation of H3K4 tri-methylation of periostin and cyclooxygenase-2 [53]. Deregulation of *AERRIE* did not result in EndMT suppression. However, we did find that JMJD2B regulates *AERRIE,* which subsequently regulates *SULF1*.

*SULF1* is an important gene involved in the migration of epithelial cells caused by liver damage [54]. There have also been findings on the role of *SULF1* in ischemic tissue repair [55]. The *SULF1* gene codes for a sulfatase that regulates TGF-β, FGF, and VEGF signaling [36,56]. Glaser et al. showed that the regulation of *SULF1* in EndMT is mediated by JMJD2B [40]. Our study shows that JMJD2B regulates *AERRIE,* and *AERRIE* in turn regulates *SULF1*. The loss or overexpression of *AERRIE* was not sufficient enough to observe an effect on mesenchymal or endothelial markers after EndMT stimulation. There are likely additional factors involved that regulate *SULF1* and subsequent EndMT.

Interestingly, JMJD2B is involved in the DNA damage pathway via ATM, STAT3, and P53 [57,58]. JMJD2B enhances double-strand break repair through the upregulation of *P53*, *P21*, *PIG3*, and *PUMA* [58]. SULF1 is found to be involved in apicidin and doxorubicin-mediated apoptosis both in vitro and in vivo [59], and promotes the proliferation of carcinoma cells [60]. A possible hypothesis to test is the involvement of SULF1 in the DNA damage pathway, to see whether the enhanced effect of apoptosis is mediated by SULF1 when endothelial cells are stimulated with HDAC inhibitor apicidin and DNA damage inducer doxorubicin. Furthermore, our previous study has shown that *AERRIE* is involved in DNA damage repair via YBX1 in endothelial cells [36]. SULF1 could be the negative feedback regulator to P53 during DNA damage response. It would be interesting to see whether JMJD2B is the DNA damage signaling regulator of *AERRIE* and *SULF1* in endothelial cells.

In this study, ECIS was performed to address the barrier function. ECIS is also a good system to examine for wound healing and migration. Due to the weak barrier of EndMT cells, electroporation of the cell layer was not performed and a different type of wound healing assay, such as the classical scratch assay, could have been a good alternative to determine the function of *AERRIE* in EndMT.

A previous study of *AERRIE* has showed its role in epithelial-to-mesenchymal transition (EMT). EMT and EndMT are described as physiological processes that are analogous to each other. Both are involved in embryonic development, but also in pathophysiological settings such as fibrosis and cancer [61,62,63]. Chung et al. describes the role of *AERRIE* as an invasion activator of EMT, mediated by activation of SNAIL in Anaplastic Large-Cell Lymphoma (ALCL) [39]. In our study, we showed the induction of *AERRIE* by EndMT, but the important EMT or EndMT transcription factor SNAIL was not regulated by *AERRIE* (Figure 2B). *AERRIE* may have a different role in EndMT compared to EMT. However, it is possible that the different uses of cytokines in both studies could induce different EndMT/EMT target genes.

The increase in mesenchymal markers indicates a loss of endothelial behavior in endothelial cells. Many studies have revealed a connection between EndMT related endothelial dysfunction, and inflammation that can result in diseases such as atherosclerosis, fibrosis, and pulmonary arterial hypertension [5,7,64]. The mechanism of the progression of these diseases by EndMT still remains unclear. A limitation of the current study is the lack of tissue experiments for the observation of *AERRIE*, *SULF1*, and *JMJD2B* expression to identify the clinical role of *AERRIE* in EndMT-related diseases. On the other hand, an in vivo model to simulate EndMT in the endothelium could give detailed information on *AERRIE*. For instance, SiO_2_ has been shown to stimulate EndMT formation in vivo with increased *COL1A1*, *Col3A1*, and *ACTA2* expression, and decreased *CDH5*, and *PECAM1* expression [65]. Additionally, a transverse thoracic-aortic constriction could be subjected to induce disturbed flow and EndMT would be subsequently induced [66]. Using these models may give us more information about the relationship between *AERRIE*, SULF1, and JMJD2B. However, we have not yet been able to identify homologues of *AERRIE* in other species, which is still the limiting factor for in vivo studies.

In conclusion, this study provides evidence that lncRNA *AERRIE* is regulated by EndMT. However, *AERRIE* is not involved in the induction of EndMT by TGF-β2 and IL-1β. Instead, *AERRIE* is required for *SULF1* expression.

## 4. Methods

### 4.1. Cell Culture

Primary human umbilical vein endothelial cells (HUVECs) were purchased from Lonza (batch p1032 and p1028) and were cultured in endothelial cell medium (ScienceCell, Carlsbad, CA, USA, 1001), supplemented with endothelial cell growth supplement (ECGS) (ScienceCell, Carlsbad, CA, USA, 1052), penicillin/streptomycin (ScienceCell, Carlsbad, CA, USA, 0503), and 5% fetal bovine serum (ScienceCell, Carlsbad, CA, USA, 0025). Primary HUVECs were cultured between passage 1 and 5 for experiments. Induction of EndMT in HUVECs was performed with IL-1β (10 ng/mL, R&D Systems, Minneapolis, MN, USA, 201-LB) and TGF-β2 (10 ng/mL, R&D Systems, 302-B2-002) for 72 h. EndMT conditions are defined as the HUVECs treated with IL-1β and TGF-β2. Hek293T cells were acquired from ATCC and cultured in Dulbecco’s Modified Eagle Medium (DMEM) (Thermo Fisher, Waltham, MA, USA, 31966021), supplemented with 10% FCS, 1% Pyruvate, 1% D-glucose, 1% penicillin/streptomycin and 1% minimum essential media with non-essential amino acid mix (Sigma-Aldrich, St. Louis, MI, USA, M7145). Hek293T and primary HUVECs were cultured at 37 °C with 5% CO_2_. Cells were counted with the Countess II cell counter (Thermo Fisher). All cell types tested negative for mycoplasma.

### 4.2. RT-qPCR

Total RNA from HUVECs was isolated with direct-zol RNA miniprep (Zymo research, Irvine, CA, USA, R2052) according to the protocol of the manufacturer, and 100–1000 ng total RNA was reverse transcribed using an iScript cDNA synthesis Kit (Bio-rad, Hercules, CA, USA, #1708891) for Real Time Quantitative PCR (RT-qPCR) analysis. The reactions were performed with iQ SYBR Green Supermix (Bio-RAD, #170-8886) in the Bio-Rad CFX96 Touch Real-time PCR system. For normalization of the samples, Ribosomal protein, large, P0 (RPLP0) was used. Gene expression analysis was done using the 2^−^^ΔΔCT^ method. Primer sequences are shown in the Supplementary Table S1.

### 4.3. SiRNAs-GapmeRs

Primary HUVECs were transfected at 50–70% confluence with 50 nM siRNAs/LNA-GapmeRs (Qiagen, Hilden, Germany) using Lipofectamine RNAiMax (Life Technologies, Carlsbad, CA, USA) according to the protocol of the manufacturer. The transfection was performed in a serum-reduced OptiMEM medium (Life Technologies). Optimem was exchanged after 4h of transfection to Endothelial Cell Medium (ECM). After 24h of stimulation with siRNAs/LNA-GapmeRs, EndMT stimulation with TGF- β2 and IL-1 β began, as described in Section 4.1. Sequences of the LNA-GapmeRs/siRNAs are listed in the Supplementary Table (Table S1).

### 4.4. Lentiviral Constructs

*AERRIE* (NR_038981.1) full-length cDNA was cloned into pLenti4/v5 (Life Technologies). Lentivirus stocks were produced in HEK293T cells using pCMVΔR8.91 as a packaging plasmid. pMD2.G (Addgene#12259) was used as vesicular stomatitis virus G glycoprotein envelope expressing plasmid [67]. Vectors with no insert were used as mock controls. Transduction was performed for 24 h.

### 4.5. Endothelial Barrier and Wound Healing

Endothelial barrier function was determined by the ECIS system (Applied BioPhysics, North Greenbush, NY, USA). 100k HUVECs were seeded per well into a gelatin-coated (1%) and L-cysteine treated 10WE plate (Applied BioPhysics). Analysis of the endothelial barrier integrity was performed after 48 h, when cells formed a stable monolayer. Barrier resistance (*R_b_*) was determined by applying an alternating current of 4000 Hz, resulting in a potential that was detected by the ECIS instrument Zθ (Applied BioPhysics), with impedance measured according to Ohm’s law. Lethal electroporation in ECIS was performed to determine cell migration and wounding.

### 4.6. Western Blot Analysis

HUVECs were lysed in Triton X-100 buffer containing benzonase (Santa Cruz Biotechnology, Cas 9025-65-4), protease inhibitors (Thermo Fisher, Halt) and phosphatase inhibitors (Thermo Fisher, Halt) for 1h on a turning wheel at 4 °C. After centrifugation with 15,000× *g*, protein content was determined with a Pierce BCA protein assay kit (Thermo Fisher). In each lane, 10 µg of protein was loaded on Sodium dodecyl sulfate (SDS) gels and blotted on 0.2 µm nitrocellulose membranes (GE healthcare, Chicago, IL, USA). As a loading control, GAPDH was used. Antibodies are shown in the Supplementary Table S2.

### 4.7. Statistical Analysis

Data are expressed as mean ± standard error of the mean (SEM). Statistical analysis was performed by Graphpad Prism 8. The data were tested using a paired or unpaired Student’s *t*-test. The Mann–Whitney test was performed when comparing two groups. Analysis of variance (ANOVA), followed by Tukey’s post-test, was performed for multiple comparisons. Statistical significance was depicted as follows: * *p* < 0.05, ** *p* < 0.01, *** *p* < 0.001, **** *p* < 0.0001, ns = not statistically significant.

## Supplementary Materials

The following are available online at https://www.mdpi.com/article/10.3390/ijms22158088/s1.
