# Supplementary material for: LncRNA AERRIE Is Required for Sulfatase 1 Expression, but Not for Endothelial-to-Mesenchymal Transition
Source: Int J Mol Sci. 2021 Jul 28;22(15):8088. doi: 10.3390/ijms22158088 (PMC8347915; doi:10.3390/ijms22158088)
Supplement: Supplementary file 1 [file ijms-22-08088-s001.zip › Supplemental tables.pdf]

Supplementary Materials

Table S1. Gene silencing and primer oligo list. Gene silencing oligos used in this study. Listed are gapmeRs to silence Aerrie and a non-targeting control gapmeR. Primers used to measure expression of the listed (human) genes by RT-qPCR.

Gene silencing oligos	Sequence (5'-3')
Gapmer ctrl	AACACGTCTATACGC
Gapmer Aerrie	GTCTTAACTGGAAGAA
Si Ctrl	MISSION siRNA Universal Negative Control #1 (SIC001)
Si JMJD2B	UGAUGCUCUCAGGGUACAG

RT-qPCR primer oligos	Sequence (5'-3')
Aerrie	AAGCCAGCATTTTTAATAAATAGGAA
	AAACTGCCTCCTCAGACTGC
RPLP0	TCGACAATGGCAGCATCTAC
	ATCCGTCTCCACAGACAAGG
SULF1	CCATTCAAGGAGGCTGCTCA
	TGTCTGCCAGTGGTTGTTGT
SNAIL	ACCACTATGCCGCGCTCTT
	GGTCGTAGGGCTGCTGGAA
CNN1	CTGAGAGAGTGGATCGAGGG
	CTGGCTGCAGCTTATTGATG
FN1	TGGTGGCCACTAAATACGAA
	GGAGGGCTAACATTCTCCAG
CTGF	GTGGAGTATGTACCGACGGC
	TCCGGGACAGTTGTAATGGC
SM22	AAGAATGATGGGCACTACCG
	ATGACATGCTTTCCCTCCTG
VE-Cadherin	CGGCGCCAAAAGAGAGATTG
	CACGCTTGACTTGATCTTGCC

Table S2. Antibody list. Antibodies used in this study. Listed are antibodies, the species these were raised in, the company these were bought from, the catolog number, the application we used these for and the used dilution.

Antibody	Species	Company	Catalog #	Application	Dilution
GAPDH	Rabbit	Cell signaling	14C10	WB	1:10000
VE-Cadherin	Rabbit	Cell signaling	2500s	WB	1:1000
SM22	Rabbit	Cell signaling	ab10135	WB	1:1000
VE-Cadherin	Rabbit	Cell signaling	2500s	IF	1:400
SM22	Rabbit	Cell signaling	ab10135	IF	1:500
anti-mouse HRP	Goat	Dako	p0447	WB	1:5000
anti-rabbit HRP	Goat	Dako	p0448	WB	1:5000
